# Isolated Primary Sinonasal Adenocarcinoma of the Sphenoid Sinus

**DOI:** 10.7759/cureus.14127

**Published:** 2021-03-26

**Authors:** Sarah Travers, Maryna Vazmitsel, Timothy Parrett, N. Scott Litofsky

**Affiliations:** 1 Division of Neurological Surgery, University of Missouri School of Medicine, Columbia, USA; 2 Department of Pathology and Anatomical Sciences, University of Missouri School of Medicine, Columbia, USA

**Keywords:** sinonsasal adenocarcinoma, sphenoid sinus lesions, ophthalmoplegia, transsphenoidal neurosurgery

## Abstract

Isolated lesions of the sphenoid sinus, particularly malignancies, are rarely reported and exist largely within the Otolaryngology literature. Delayed diagnosis may necessitate neurosurgical involvement; therefore, neurosurgeons must be aware of the range of pathologies in this region in order to provide adequate treatment. We present an unusual case of an 89-year-old female with several weeks of worsening headaches, vision loss, and cranial neuropathies. Work-up at an outside hospital was non-diagnostic. After referral, an expansive and erosive lesion within the left sphenoid sinus was identified. A transsphenoidal approach for resection of the lesion yielded a primary non-salivary non-intestinal type sinonasal adenocarcinoma, as well as bacterial sinusitis and probable allergic fungal sinusitis. The patient was treated with antimicrobial medications as well as stereotactic radiosurgery. Her neurological deficits did not improve with treatment, and she ultimately expired 3.5 months post-operatively after transition to hospice. Primary sinonasal adenocarcinoma is a very rare pathology in this location. Surgical intervention is necessary to obtain an accurate diagnosis and proceed with appropriate treatment. Delayed diagnosis likely portends a worse prognosis.

## Introduction

Isolated lesions of the sphenoid sinus are rarely reported; most of these few cases have been described in the Otolaryngology literature. Primary malignancies in this location are particularly infrequent but can have irreversible consequences when overlooked in the differential diagnosis. Because of the location and symptoms associated with these lesions, neurosurgeons may not become involved in the patient’s care. On rare occasion, however, neurological deficits may develop late in the disease course, precipitating a request for neurosurgical assessment; therefore, neurosurgeons should be aware of the range of pathologies associated with this location. Here, we report the unusual case of a primary non-salivary gland non-intestinal type sinonasal adenocarcinoma located within the sphenoid sinus associated with ophthalmoplegia and visual loss.

## Case presentation

**Figure 1:**
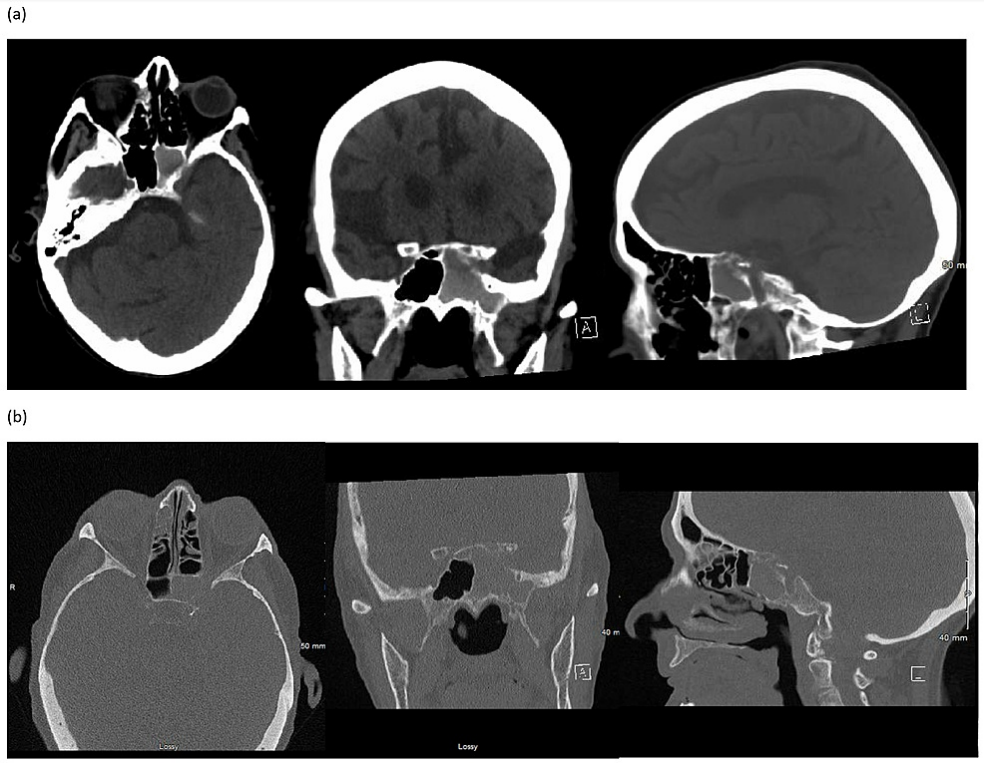
Axial, coronal, and sagittal images from (A) CT head and (B) CT sinus imaging showing opacification of left sphenoid sinus with bony expansion and erosion of the lateral wall

The patient was an 89-year-old woman with a recent history of urothelial carcinoma who presented with worsening headaches and left-eye vision loss over the previous four weeks. While her headaches had been present for several months, the vision loss began a couple of weeks following an intravitreal injection for macular degeneration. She had been following with her outside ophthalmologist who suspected temporal arteritis; however, temporal artery biopsy was negative, and steroids failed to improve her symptoms. A CT head obtained at an outside facility was reportedly negative though unavailable for our review, and because of an incompatible pacemaker, she was unable to have an MRI. She was admitted initially to the Neurology service for workup of stroke versus malignancy, with lumbar puncture returning with a normal CSF profile and cytology negative for malignant cells. Ophthalmology evaluation revealed essentially complete left eye ophthalmoplegia with an unreactive left pupil. CT angiography performed on their recommendation revealed a hyperdensity within the left sphenoid sinus, as well as erosion of the supralateral wall, extension into the optic canal, and enhancement of the left cavernous sinus, concerning initially for an infectious process (Figure 1A). At this time, Otolaryngology and Neurosurgery were both consulted, and a CT sinus was then performed (Figure 1B). Otolaryngology performed a bedside flexible endoscopic evaluation of the nasal sinuses, which did not reveal any sign of infection or malignancy; therefore, in order to obtain a tissue diagnosis, the patient was taken to the operating room with Neurosurgery for a microscopic sublabial approach to the sphenoid sinus. Upon entering the sinus, a hemorrhagic lesion resembling clotted blood was encountered which was collected for a specimen. The mucosa was stripped and sent for a specimen as well. No obvious bony defect was visualized under microscopic evaluation, but the surface of the superio-lateral sphenoid sinus was irregular. The frozen section in the operating room reported acute and chronic sinusitis. The patient remained clinically stable but unfortunately did not have any improvement in her vision or extraocular movements post-operatively.

**Figure 2:**
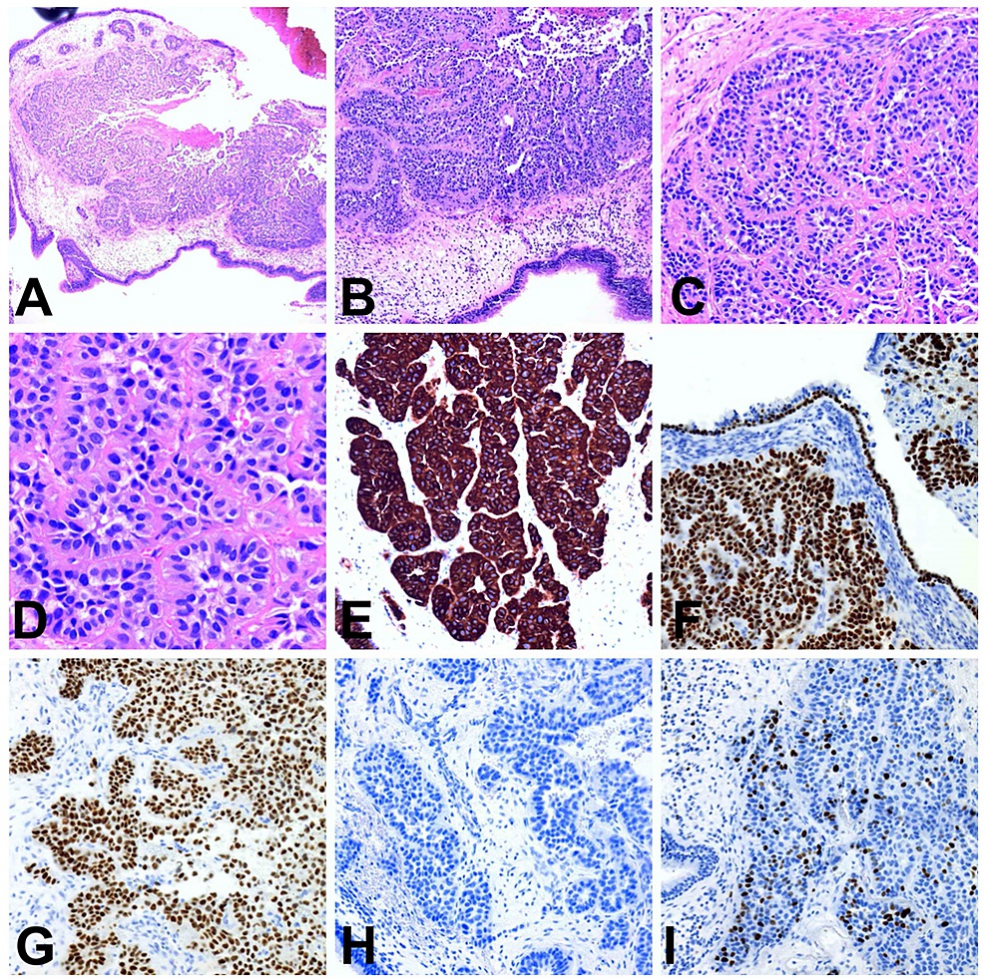
(A) Sphenoidal submucosal tumor at scanning magnification; note the absence of mucosal ulceration, necrosis, or desmoplastic stromal change (H&E stain, 2x5). (B) At low magnification, the tumor is characterized by tubular-papillary growth pattern (H&E stain, ×40). (C) Tubular-papillary structures with a single layer of cuboidal to columnar neoplastic cells with nuclear stratification and absence of a delimiting myoepithelial cell layer (H&E stain, ×100). (D) Closer view of neoplastic cells reveals bland low-grade cytology with minimal nuclear atypia: cells have moderate to scant amount of pale eosinophilic cytoplasm, round uniform nuclei, and indistinct nucleoli. Mitotic figures are not visible (H&E stain ×200). (E) Neoplastic cells are strongly CK7 immunopositive (×100). The nuclear expression of p63 (F) and GATA-3 (G) in neoplastic cells (×100). (H) The absence of synaptophysin expression in tumor cells (×100). (I) Ki67 index 10% in this low power field (×100).

The surgical pathology specimen was examined by our neuropathology team and independently reviewed by an outside pathologist specializing in otolaryngic pathology. The left sphenoid lesion was fixed in formalin, embedded in paraffin, and examined on four-micron sections stained with H&E. Immunoperoxidase studies with appropriate controls were performed with the following antibodies: CK7, CK20, CK5/6, CDX2, b-catenin, TTF1, p63, p16, p53, SOX-10, GATA3, S100, synaptophysin, chromogranin, estrogen receptors (ER), AMACR, and Ki67. Microscopically, the tumor was located growing beneath non-ulcerated mucosa, and the complexity of the growth pattern and local invasive growth supported the diagnosis of malignancy. The morphological features were consistent with sinonasal non-salivary gland non-intestinal type adenocarcinoma, although the immunophenotype supported this diagnosis only partially. There was no evidence of SOX10 and S100 expression that is characteristic of this entity. At the same time, the absence of CDX2 and CK20 immunoreactivity helped to rule out sinonasal non-salivary gland intestinal-type adenocarcinoma, and the lack of TTF1 expression ruled out a metastatic lung and thyroid carcinoma. Figures 2A-2I describe the surgical pathology findings in further detail.

Although no definitive fungal elements were identified on the nasal biopsy, there were features consistent with this additional diagnosis including the presence of eosinophilic mucus, eosinophils, and Charcot-Leyden crystals. Culture results eventually returned positive for methicillin-sensitive Staphylococcus aureus; therefore, she was treated with two weeks of augmentin and posconazole. After a multidisciplinary discussion with our Hematology and Radiation Oncology teams, she received outpatient palliative radiation of 2000cGy in five fractions to the left sphenoid sinus and cavernous sinus. She was not felt to be a good candidate for chemotherapy given her age and evidence of renal and spinal metastases on positron emission tomography (PET) imaging. At the last follow-up, three weeks after surgery, the patient’s neurologic deficits were stable. She chose to transition to hospice shortly after that appointment in order to avoid lengthy travel to our institution and further diagnostic studies, which she did not feel would improve her outcome. The patient died 3.5 months after surgery.

## Discussion

Only a handful of reports of primary adenocarcinoma of the sphenoid sinus are described in the literature [1-5]. According to two reviews of a combined 314 patients with isolated sphenoid sinus lesions, the vast majority of pathologies found in this location are inflammatory, including sinusitis, mucocele, and fungal disease, while primary malignancy accounted for only 11.3%-13.1% of the total [3,6]. Of these, squamous cell carcinoma occurs most frequently [3-5,7], with presenting symptoms most commonly including headaches, visual disturbance, and diplopia [3,4,6]. Sinonasal carcinomas, including adenocarcinoma, have a male predilection [3,8] and are associated with occupational exposures, particularly wood dust [8].

Radiographic imaging is the diagnostic tool of choice, with key CT findings in malignancy being bony destruction or erosion [3,4,7,8]. MRI is useful for differentiating tumor from obstructed secretions and evaluating for soft tissue and intracranial spread [7]. The staging of cancers of the paranasal sinuses is described in the 8th edition of the American Joint Committee on Cancer (AJCC) Cancer Staging Manual and is defined by the TMN staging system [7]. Tumors extending beyond mucosa of the sinus (T1) are considered higher stage when causing bony erosion (T2), invasion of surrounding structures or sinuses (T3), or advanced local extension (T4), particularly when combined with lymph node involvement or distant metastases [7]. While surgery, chemotherapy, and radiation are all options for treatment, advanced-stage tumors carry a poor prognosis given the difficulty in achieving a surgical cure and variable sensitivity to adjuvant treatments [3,4]. Five-year survival rates for those with regional or distant spread of disease remain under 20% for those receiving no treatment [5], and the worse outcome is associated with age above 60 and advanced tumor grade/stage [9,10]. However, survival advantages have been identified in patients with localized disease receiving treatment [5], patients without cranial neuropathies [4], and patients receiving surgery and radiation [9], stressing the importance of timely evaluation and workup.

This case particularly demonstrates the importance of obtaining a tissue sample for appropriate diagnosis and treatment. Presumptive treatment for sphenoid sinusitis based on the general prevalence of infection in this region and the radiology impression of sinusitis in this patient’s pre-operative imaging would have undoubtedly allowed even further spread of the tumor. The patient ultimately required two different treatments for separate pathologies, which would not have been detected without a tissue diagnosis. Additionally, it was necessary to exclude a more invasive fungal sinusitis such as mucormycosis, which carries significant morbidity and mortality. The authors chose a microscopic approach to the sphenoid sinus based on preferred surgeon experience. The endoscopic approach may have allowed better visualization of the suspected region of erosion into the cavernous sinus, although this would likely not have changed the patient’s outcome, and previous research indicates that microscopic surgery of the paranasal sinuses is a safe and effective alternative to endoscopic surgery [11]. Additionally, in our institution, total surgery and anesthesia time is shorter for an open sublabial approach, and the length of hospital stay is comparable to endoscopic surgery.

## Conclusions

While isolated lesions of the sphenoid sinus are most likely to be infectious, physicians should include malignancy on the list of differential diagnoses, particularly in patients presenting with vision loss and cranial neuropathies. CT imaging should include careful study of the extracranial structures and should evaluate for bony expansion or erosion which could indicate malignancy. Referral to a tertiary care center should be initiated early in the disease course in those with neurologic signs and symptoms. Tissue diagnosis is necessary to confirm the pathology which can be performed through a microscopic or endoscopic approach. Surgery, chemotherapy, and/or radiation are all treatment options based on the stage of the disease, with earlier detection and diagnosis being key to a more favorable prognosis.
